# Roles of type II H^+^-PPases and PPsPase1/PECP2 in early developmental stages and PPi homeostasis of *Arabidopsis thaliana*

**DOI:** 10.3389/fpls.2023.1031426

**Published:** 2023-01-27

**Authors:** Hiroshi Tojo, Hiromitsu Tabeta, Shizuka Gunji, Masami Y. Hirai, Pascale David, Hélène Javot, Ali Ferjani

**Affiliations:** ^1^ Department of Life Sciences, Graduate School of Arts and Sciences, The University of Tokyo, Tokyo, Japan; ^2^ Department of Biology, Tokyo Gakugei University, Koganei, Tokyo, Japan; ^3^ RIKEN Center for Sustainable Resource Science, Yokohama, Japan; ^4^ Aix Marseille Univ, CEA, CNRS, BIAM, Saint Paul-Lez-Durance, France; ^5^ Aix Marseille Univ, CEA, CNRS, BIAM, Marseille, France

**Keywords:** *Arabidopsis thaliana*, pyrophosphate homeostasis, vacuolar type-I H^+^-PPase, vacuolar type-II H^+^-PPases, PPsPase1/PECP2, metabolism

## Abstract

The regulation of intracellular pyrophosphate (PPi) level is crucial for proper morphogenesis across all taxonomic kingdoms. PPi is released as a byproduct from ~200 metabolic reactions, then hydrolyzed by either membrane-bound (H^+^-PPase) or soluble pyrophosphatases (PPases). In Arabidopsis, the loss of the vacuolar H^+^-PPase/FUGU5, a key enzyme in PPi homeostasis, results in delayed growth and a number of developmental defects, pointing to the importance of PPi homeostasis in plant morphogenesis. The Arabidopsis genome encodes several PPases in addition to FUGU5, such as PPsPase1/PECP2, VHP2;1 and VHP2;2, although their significance regarding PPi homeostasis remains elusive. Here, to assess their contribution, phenotypic analyses of cotyledon aspect ratio, palisade tissue cellular phenotypes, adaxial side pavement cell complexity, stomatal distribution, and etiolated seedling length were performed, provided that they were altered due to excess PPi in a *fugu5* mutant background. Overall, our analyses revealed that the above five traits were unaffected in *ppspase1/pecp2*, *vhp2;1* and *vhp2;2* loss-of-function mutants, as well as in *fugu5* mutant lines constitutively overexpressing *PPsPase1/PECP2*. Furthermore, metabolomics revealed that *ppspase1/pecp2*, *vhp2;1* and *vhp2;2* etiolated seedlings exhibited metabolic profiles comparable to the wild type. Together, these results indicate that the contribution of PPsPase1/PECP2, VHP2;1 and VHP2;2 to PPi levels is negligible in comparison to FUGU5 in the early stages of seedling development.

## Introduction

Pyrophosphate (PPi) is produced from nearly 200 different metabolic reactions, as a byproduct of macromolecule synthesis (Heinonen, 2001). It is released during the production of DNA, RNA, proteins and polysaccharides, and can reciprocally be used as a phosphate donor or an energy donor in conditions where ATP levels are limited, although this dogma was challenged recently (Wimmer et al., 2021). Due to the reversibility of many enzymatic reactions, PPi overaccumulation is strictly avoided in the plant cytoplasm, in order to prevent retroinhibition of metabolic pathways (Stitt, 1998; Wimmer et al., 2021). PPi-degrading enzymes (i.e. pyrophosphatases) are key players in the maintenance of the PPi cell concentration, and belong to very distinct families of soluble or membrane-bound proteins (Heinonen, 2001).

**Figure 1:**
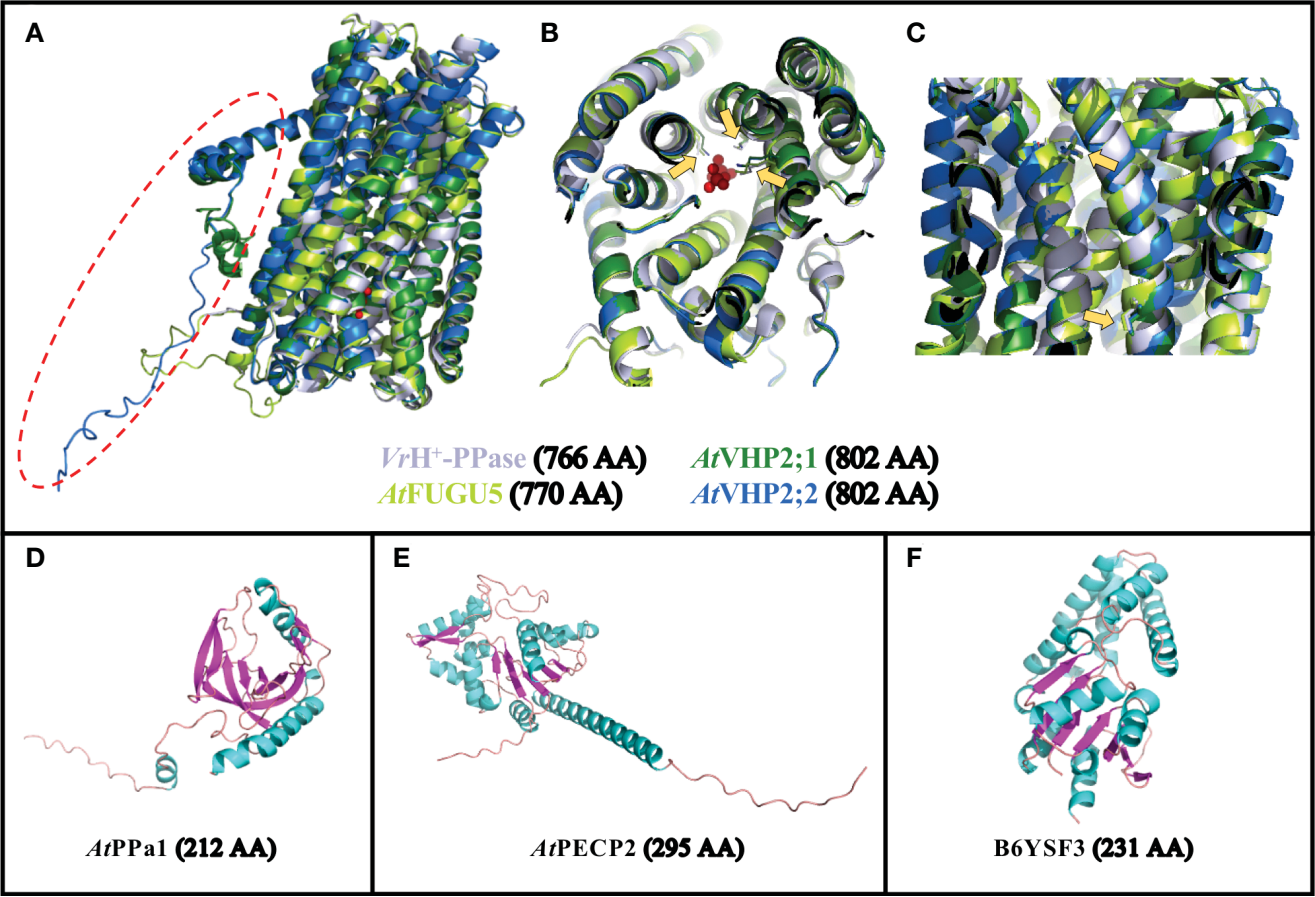
Predicted structures of selected PPases. **(A–C)** Overlay of the predicted structures for FUGU5 (UniProt P31414; in bright green),VHP2;1 (UniProt Q56ZN6; in dark green) and VHP2;2 (UniProt Q9FWR2; in blue) onto the crystal structure of *Vr*H^+^-PPase (PDB reference 4A01, Chain A; in light gray). **(A)** General overview of the 4 structures showing the strong conservation of these 16-TM proteins, with the exception of an additional N-terminal domain in VHP2;1 and 2;2 (surrounded by a red dotted circle). **(B)** Rotated view showing the positions of 3 AA (yellow arrows) involved in PPi binding in *Vr*H^+^-PPase, overlapping with the corresponding positions within the 3 predicted structures of FUGU5, VHP2;1 and VHP2;2. **(C)** Side view showing the positions of 2 AA (yellow arrows) involved in proton pumping in *Vr*H^+^-PPase, overlapping with the corresponding positions within the 3 predicted structures of FUGU5, VHP2;1 and VHP2;2. **(D)** Predicted structure for PPa1 (UniProt Q93V56). **(E)** Predicted structure for PECP2 (UniProt Q67YC0). **(F)** Predicted structure for *T. onnurineus* TON_0002 PPase (UniProt B6YSF3). The number of AA for the full-length proteins is indicated between brackets. AA, amino acid.

The vacuolar (type-I) V-PPases are 16 TM-domain proteins, and are the most well-described category of pyrophosphatases. The crystal structure of the V-PPase from *Vigna radiata* (*Vr*H^+^-PPase) has been resolved (**Figure 1A**; **Supplemental Movie 1**), revealing residues involved in PPi hydrolysis (**Figure 1B**; **Supplemental Movie 1**) and proton transport (**Figure 1C**; **Supplemental Movie 1**; Lin et al., 2012; Tsai et al., 2019). In Arabidopsis, this family is represented by a single gene (*FUGU5*/*VHP1*;*1*/*AVP1*/AT1G15690; **Figures 1A–C**). The study of V-PPase loss-of-function *fugu5* mutants has revealed the importance of this enzyme, as they display a range of mild phenotypic defects that are particularly clear in tissues showing intense gluconeogenesis activity (growing tissues and etiolated seedlings in particular). FUGU5 is a bifunctional protein that also combines proton pumping and PPase activities (**Figures 1B, C**), and is capable of translocating protons from the cytosol into the vacuole lumen by PPi hydrolysis (Maeshima, 2000). The consequences of altering PPase activity in *fugu5* mutants are particularly clear for germinating seedlings, and this phenotype is independent from FUGU5 proton pumping activity (Bertoni, 2011; Ferjani et al., 2011; Asaoka et al., 2016; Takahashi et al., 2017). Increased PPi levels in *fugu5* are associated with inhibited hypocotyl elongation in etiolated seedlings (Ferjani et al., 2011). Seedlings grown in the light also display an altered cotyledon shape, as well as modifications of their palisade tissues, adaxial-side pavement cell complexity and stomatal distribution (Ferjani et al., 2007; Ferjani et al., 2011; Asaoka et al., 2019; Fukuda et al., 2020; Gunji et al., 2020; Gunji et al., 2022). These characteristics can be rescued by expressing IPP1, a soluble yeast pyrophosphatase (Ferjani et al., 2011; Segami et al., 2018; Gunji et al., 2020; Gunji et al., 2022). The proton pumping activity of FUGU5 is important in conditions where ATP levels are limiting, allowing the cells to sustain a proton gradient between the vacuole and the cytosol even when the primary vacuolar proton pumps (V-ATPases) display limiting activities, such as during anoxia (Maeshima, 2000; Kriegel et al., 2015). The remarkable abundance of these two categories of proton pumps (V-PPases and V-ATPases) within the vacuolar membrane highlights the importance of maintaining an active proton gradient (ΔpH) in a wide range of conditions (Gaxiola et al., 2007; Schumacher, 2014; Kriegel et al., 2015).

Other proteins are capable of degrading PPi in plant cells, which explains why *fugu5* mutants only show phenotypic defects in conditions where PPi production is particularly intense (Ferjani et al., 2011; Ferjani et al., 2014). A number of proteins use PPi as a cofactor to empower metabolic reactions, such as the enzyme pyrophosphate fructose-6-phosphate 1-phosphotransferase (PFP or PPi-PFK; EC 2.7.1.90), which is responsible for the reversible conversion of fructose-6-phosphate into fructose-1,6-biphosphate (Lim et al., 2009; Lim et al., 2014; Duan et al., 2016). In addition, UDP-glucose pyrophosphorylase (UGPase; EC 2.7.7.9) catalyzes the reversible production of UDP-Glu and PPi from a range of substrates (Kleczkowski et al., 2004; Liu et al., 2021). Clearly, their enzymatic activities are not centered on PPi homeostasis, although their ability to use PPi as a substrate and their sensitivity to PPi levels does explain why impairing PPi homeostasis has such a strong impact on carbohydrate metabolism.

In contrast to these enzymes, soluble pyrophosphatases (sPPases; **Figure 1D**) are dedicated to the degradation of PPi into two Pi molecules (Öztürk et al., 2014; Gutiérrez-Luna et al., 2016; Segami et al., 2018). The crystal structure of soluble AtPPa1 was recently determined, and its strong similarity to yeast IPP1 sPPase was confirmed (Grzechowiak et al., 2019). Their comparison highlighted the highly conserved active sites and their organization, but also revealed that PPa1 is even closer to prokaryotic PPases (Grzechowiak et al., 2019). PPa1 enzyme is composed of nine β-strands and two α-helices, with a compact core containing five anti-parallel β-barrels (**Figure 1D**; Grzechowiak et al., 2019). In Arabidopsis, sPPases are mostly targeted to the cytosol (AtPPa1 through 5), whereas only one is targeted to the plastids (AtPPa6; Öztürk et al., 2014; Schulze et al., 2004). Previous work has shown that mutations in these enzymes could have strong developmental defects (hypocotyl elongation, cell wall composition, starch content, etc.), particularly when combined with *fugu5* mutations (Segami et al., 2018). The severity of the phenotype was then correlated with the level of PPi increase in the cell. The large set of sPPase genes, together with the abundance of V-PPase in the vacuolar membrane, is another indication of the tight control exerted by plant cells on PPi cytoplasmic levels (Kriegel et al., 2015; Segami et al., 2018).

In addition to these different categories of PPases, several other plant pyrophosphatases are associated with subcellular compartments. Type-II H^+^-PPases (VHP2/AVP2 or AVPL1-like) have a dual function of PPi hydrolysis and H^+^-translocation (Drozdowicz and Rea, 2001), like the vacuolar FUGU5, however they are localized in the Golgi (Drozdowicz and Rea, 2001; Mitsuda et al., 2001; Segami et al., 2010). This family contains two genes in Arabidopsis, *AtVHP2;1* (At1g78920) and *AtVHP2;2* (At1g16780), in which *VHP2;1* expression levels are much higher than *VHP2;2* levels (Segami et al., 2010). *VHP2;1* is particularly expressed in the cotyledons and roots of young seedlings, as well as in the trichomes of rosette leaves, and the stamina (Mitsuda et al., 2001). Type-I and type-II H^+^-PPases show highly conserved structures, with the exception of an additional N-terminal domain for type-II PPases (**Figures 1A–C**; **Supplemental Movie 1**); however, type-II H^+^-PPases are expressed at much lower levels than type-I H^+^-PPases (Segami et al., 2010). Although the properties and expression patterns of these PPases have been well-studied (Drozdowicz and Rea, 2001; Segami et al., 2010), their *in vivo* significance regarding their physiological role remains to be characterized. The precise overlap of their structures with type-I H^+^-PPase (**Figure 1**) reflects their dual function in PPi hydrolysis and proton pumping, driving Golgi acidification. However, the importance of this protein in terms of overall cytosolic PPi homeostasis within the cell, and in terms of Golgi acidification, still remains to be discovered.

The endoplasmic reticulum compartment might be associated with a final PPase category: Arabidopsis HAD-type protein PPsPase1/PECP2 (encoded by At1g73010; **Figure 1E**). This protein was identified based on its fast responsiveness to phosphate starvation (May et al., 2011; Hanchi et al., 2018; Angkawijaya et al., 2019). Phosphate is an essential macronutrient for plant growth and development, and its deficiency triggers striking morphological changes in plant roots, such as reduced primary root length and increased lateral root length, as well as root hair length and density (Williamson et al., 2001; Linkohr et al., 2002; Al-Ghazi et al., 2003; Reymond et al., 2006). From a structural point of view, PPsPase1/PECP2 is completely different from sPPases (**Figures 1D, E**), as it possesses the typical structure of HAD hydrolases, composed of a Rossmann-like fold (a set of about 6 parallel β-strands surrounded by additional helices on each side), completed by an additional “cap domain” (Kuznetsova et al., 2015). The enzymatic characterization of the recombinant protein PPsPase1/PECP2 in *Escherichia coli* revealed an efficient cleavage of PPi by this enzyme, making it one of the rare pyrophosphatases from the HAD superfamily, along with others including the first-identified member (B6YSF3; **Figure 1F**) from *Thermococcus onnurineus* (Lee et al., 2009; May et al., 2011). Another enzyme among the rare HAD family members with pyrophosphatase activity is BT2127 from *Bacteroides thetaiotaomicron*, which has been crystallized (Huang et al., 2011). The His23 and Lys79 residues of BT2127 are thought to be responsible for the efficient PPi catalysis of this protein, whereas most of the other HAD members show a phosphomonoesterase activity (Zhang et al., 2022). As these two residues do not seem to be conserved in B6YSF3 (data not shown), it appears that this mechanisms might not be a universal feature of PPases belonging to the HAD family. In the case of PECP2, the sequences are even more divergent from the above two reference HAD-type PPases. Experiments with *ppspase1/pecp2* mutants, especially when combined with mutations in the homolog gene *pecp1*, revealed that the most obvious phenotype was related to altered phosphocholine (PCho) and phosphoethanolamine (PEtn) content (Hanchi et al., 2018; Tannert et al., 2018; Angkawijaya and Nakamura, 2017; Angkawijaya et al., 2019). Since PECP1 exhibits an affinity for PCho and PEtn when expressed in *E. coli* (May et al., 2012), it was suggested that PPsPase1/PECP2 might actually favor this substrate *in vivo* rather than PPi. Indeed, KO mutants of *PPsPase1/PECP2* did not display any phenotypes that are classically associated with PPi defects (Hanchi et al., 2018). In addition, the combination of *fugu5* with *ppspase1*/*pecp2* and *pecp1* mutations did not accentuate *fugu5* phenotypes. However, it remains to be demonstrated if *ppspase1*/*pecp2* has any detectable impact on PPase homeostasis *in planta*. A third and final homolog named *ThMPase1/PECP3* (encoded by At4g29530) is expressed in plants at low levels, and does not respond to Pi availability (Hanchi et al., 2018; Tannert et al., 2021). Based on heterologous expression in yeast, this homolog was initially shown to dephosphorylate thiamine monophosphate with a high efficiency (Hasnain et al., 2016). PEtn was later revealed to be the favored substrate of this enzyme *in planta*, instead of thiamine monophosphate (Tannert et al., 2021).

Clearly, there is a need to further characterize *VHP2;1, VHP2;2* and *PPsPase1*/*PECP2* in relation to their physiological functions; moreover, the unambiguous demonstration of a substantial impact on overall PPi content *in planta* is lacking. Although the quantification of PPi levels in KO lines might be a useful strategy, PPi is notoriously difficult to extract from plant cells for quantification due to its labile nature (Heinonen, 2001). Analysis of cell contents by ion-exchange chromatography (Yoza et al., 1991; Ruiz-Calero and Galceran, 2005) was instrumental in demonstrating the altered PPi content of several PPi homeostasis mutants (Ferjani et al., 2011; Segami et al., 2018), but PPi quantification from plant extracts remains rare. Nevertheless, there is an active field of research focused on developing methods for *in vivo* PPi measurements based on fluorescent probes. Although recent progress in this domain seems promising (Wang et al., 2021), no probe has yet been established as a standard.

As a proxy for direct PPi quantification, one powerful substitute consists in revealing alterations of PPi homeostasis based on plant phenotypic changes (Ferjani et al., 2011; Segami et al., 2018; Asaoka et al., 2019; Fukuda et al., 2020; Gunji et al., 2020; Tabeta et al., 2021). Thanks to the in-depth characterization of Arabidopsis mutants showing altered PPi homeostasis, specific plant anatomical parameters can be quantified to reveal subtle alterations in parameters linked to PPi concentration.

The *fugu5* mutants were initially characterized on the basis of the alteration of their cotyledon shape (Ferjani et al., 2007). Later, it was revealed that altered PPi levels were also responsible for hypocotyl elongation defects in etiolated seedlings (Ferjani et al., 2011). This parameter, more easily quantifiable than the determination of cotyledon rotundity, is inversely correlated with PPi accumulation in germinating seedlings grown in the absence of sucrose (Segami et al., 2018). This phenotype is strongly aggravated when combined with a mutation in the cytosolic sPPase *PPa1* gene, whereas combination with *ppa2* through *5* did not enhance the *fugu5* phenotype. Using *fugu5 ppa1* double mutants was necessary to validate the *in vivo* PPase activity of PPa1, since the effects of a single *ppa1* mutation can be masked by the activity of the vacuolar (type-I) FUGU5 V-PPase (Segami et al., 2018; Fukuda et al., 2020). Combinations of *fugu5* and candidate PPases are therefore capable of revealing the importance of genes in relation to PPi homeostasis, in a highly quantifiable manner. Other parameters such as cell complexity or cell wall composition were subsequently identified as also altered in the *fugu5* mutant in response to PPi levels (Segami et al., 2018; Fukuda et al., 2020; Gunji et al., 2020; Gunji et al., 2022). Altogether, the quantification of these parameters makes it possible to unambiguously predict a PPi homeostasis defect for novel mutants.

Reciprocally, it is also possible to demonstrate the PPase activity of enzymes by overexpressing candidate genes in a *fugu5* mutant background, as shown with the heterologous expression of the soluble yeast pyrophosphatase IPP1 in Arabidopsis, which rescued the *fugu5* phenotype (Ferjani et al., 2011; Asaoka et al., 2019; Gunji et al., 2020; Gunji et al., 2022). This complementation also demonstrated that the developmental alterations of *fugu5* were due to the loss of pyrophosphatase function, rather than its proton translocation activity. This approach is similar to the complementation of the growth defect of yeast deficient in the endogenous cytosolic PPase (IPP1), which can be complemented by overexpression of *IPP1* or other PPases, as previously demonstrated for Arabidopsis sPPases (Schulze et al., 2004; Segami et al., 2018). Altogether, the use of *fugu5* as a standard instead of WT plants, combined with the scoring of anatomical properties, makes it possible to detect increases in cytosolic PPi levels even beyond the ones in *fugu5* (as reported in Segami et al., 2018), or a range of intermediate levels between those in WT and *fugu5* (as reported in Ferjani et al., 2011).

Here, to assess the contribution of type-II H^+^-PPases (VHP2;1, VHP2;2) and PPsPase1/PECP2 to PPi homeostasis, we opted for an in-depth phenotypical characterization of single and combined mutants, as well as a set of overexpressing lines. As we show that no obvious alteration of the overall PPi levels is associated with PPsPase1/PECP2, we use the name PECP2 throughout the manuscript. In addition, our study presents metabolomics data that provide a complete overview of the mutant characterizations, focusing on the early stages of seedling development.

## Materials and methods

### Comparison of protein structures

The structures of the full length proteins corresponding to FUGU5 (UniProt Accession P31414), VHP2;1 (UniProt Q56ZN6), VHP2;2 (UniProt Q9FWR2), PPa1 (UniProt Q93V56), PECP2 (UniProt Q67YC0) and *Thermococcus onnurineus* TON_0002 PPase (UniProt B6YSF3) were predicted using AlphaFold (Jumper et al., 2021; Varadi et al., 2022). The crystal structure from *Vr*H^+^-PPase (UniProt 21616) was obtained from the Protein Data Bank (PDB reference 4A01, Chain A; Lin et al., 2012). Overlays and visualization of structures were performed using The PyMOL Molecular Graphics System, Version 2.5 Schrödinger, LLC.

### Plant materials and growth conditions

Mutants and all transgenic plants were in the Columbia-0 (Col-0) background, and *Arabidopsis thaliana* Col-0 was used as the wild type (WT). *fugu5-1* and *fugu5-3* were isolated and characterized as the loss-of-function mutant of the vacuolar type-I H^+^-PPase (Horiguchi et al., 2006b; Ferjani et al., 2007; Ferjani et al., 2011; Fukuda et al., 2016; Segami et al., 2018; Fukuda et al., 2020; Gunji et al., 2020; Gunji et al., 2022). *pecp2-1* and *pecp2-3* (previously described as *ppspase1-1* or *ps2-1*, and *ppspase1-3*, respectively), *pecp1-1*, the *fugu5-1 pecp2-1 pecp1-1* triple homozygous mutant (Hanchi et al., 2018), and the *vhp2;1* and *vhp2;2-2* (Segami et al., 2010) loss-of-function mutants were previously reported.

Seeds were sown on rockwool (Nippon Rockwool Corp.), watered daily with 0.5 g L^−1^ Hyponex solution and grown under a 16/8-h light/dark cycle with white light from fluorescent lamps at approximately 50 μmol m^−2^ s^−1^ and 22°C.

Sterilized seeds were sown on sucrose-free Murashige and Skoog (MS) medium (Wako Pure Chemical). 0.1% (w/v) 2-(N-morpholino) ethanesulfonic acid (MES) was added, the pH was adjusted to 5.8 with KOH, and then the medium was solidified using 0.2% to 0.5% (w/v) gellan gum (Murashige and Skoog, 1962). The seeds were sown on the MS plates, which were then stored at 4°C in the dark for 3 days of cold treatment. After cold treatment, the seedlings were grown either in the light (as indicated above) or in the dark for the indicated periods of time.

### Generation of transgenic plants and gene overexpression quantification by RT-qPCR

To overexpress *PECP2* under *35S* promoter control, *fugu5-1* and *fugu5*-*3* mutants (Ferjani et al., 2011) were transformed with the previously described *Prom35S::PECP2* plasmids (Hanchi et al., 2018). Plant transformation was performed using *Agrobacterium tumefaciens* C58C1 with a simplified version of the floral dip method (Clough and Bent, 1998; Logemann et al., 2006).

After transformation, T_1_ transformants were selected on soil using Basta, and a preliminary selection of overexpressing lines was performed by RT-qPCR. Only overexpressing lines showing a 3:1 segregation of PPT resistance in T_2_ (suggesting a single T-DNA insertion event) were re-amplified to obtain homozygous plants in T_3_. Quantification of the overexpression level was performed by RT-qPCR in the T_3_ generation as in Hanchi et al. (2018), using *ROC3* and *GAPC1* as reference genes. RNA was extracted from two independent biological replicates (9-12 plants; 50–90 mg of frozen plant tissue per sample). Data were analyzed using the Relative Expression Software Tool REST2009 running 5,000 iterations of the randomized test (Pfaffl et al., 2002).

### Morphological observations and cellular phenotypic analyses

Photographs of gross plant phenotypes at 10 days after seed sowing (DAS) and 3 days after induction of seed germination (DAI) were obtained using a stereoscopic microscope (M165FC; Leica Microsystems) connected to a charge-coupled device (CCD) camera (DFC300FX; Leica Microsystems). Cotyledons were fixed in formalin–acetic acid–alcohol (FAA; 4% formalin, 5% acetic acid, and 50% ethanol) and cleared with chloral solution (200 g of chloral hydrate, 20 g of glycerol, and 50 mL of deionized water) to measure cotyledon area and cell number, as previously described (Tsuge et al., 1996). Whole cotyledons were observed using a stereoscopic microscope equipped with a CCD camera. Cotyledon palisade tissue cells were observed and photographed under a light microscope (DM-2500; Leica Microsystems) equipped with Nomarski differential interference contrast optics and a CCD camera. Cell size was determined as mean palisade cell area, determined from a paradermal view, as previously described (Ferjani et al., 2011). The cotyledon aspect ratio was calculated as the ratio of the cotyledon blade length to its width.

### Electron microscopy observation and quantitative analysis of epidermal cells

For scanning electron microscopy (SEM), cotyledons were dissected from plants at the indicated growth stages. Samples were fixed overnight in FAA at room temperature. The fixed specimens were dehydrated in an ethanol series (50, 60, 70, 80, 90, 95, 99.5, and 100% [v/v]; 60 min per step) and stored overnight in 100% (v/v) ethanol at room temperature. The ethanol was replaced with 3-methylbutyl acetate and the samples were dried in a critical-point dryer (JCPD-5; JEOL), sputter-coated with gold–palladium using an anion sputter (JFC-1100; JEOL), and examined under an S-3400N SEM (Hitachi), as previously described (Maeda et al., 2014; Gunji et al., 2020; Gunji et al., 2022). SEM images of the adaxial side of cotyledons were used to quantify pavement cell complexity. The area and perimeter of individual pavement cells (25 cells from one cotyledon; six cotyledons in total) were measured using ImageJ (version 1.63), and their complexity was quantified by calculating the undulation index (UI; Thomas et al., 2003) using the following equation (Kürschner, 1997):


$$UI=\frac{Ce}{2\pi \sqrt{Ae/\pi }}$$

where UI (dimensionless) is the undulation index, *Ce* (µm) is the cell perimeter, and *Ae* (µm^2^) is the cell area. Note that an increased undulation index means increased pavement cell complexity, and *vice versa*. The stomatal index (SI) is the percentage of the number of stomata to the total number of epidermal cells. The SI was calculated using the following equation:


SI = (St x 100)/(E + St)

where St is the number of stomata per unit area, and E is the number of epidermal cells within the same unit area.

### Wide-target metabolome analysis of GC-QqQ-MS

A total of 100 etiolated seedlings at 3 DAI were collected in one tube in liquid nitrogen and freeze-dried. Subsequently, the samples were extracted using a bead shocker in a 2-mL tube with 5-mm zirconia beads and 80% MeOH for 2 min at 1,000 rpm (Shake Master NEO, Biomedical Sciences). The extracted solutions were centrifuged at 1,000 rpm for 1 min, and 100 µL of centrifuged solution and 10 µL of 0.2 mg/mL Adonitol (Internal Standard; I.S.) were dispensed into 1.5-mL tubes. After drying the solution using a centrifuge evaporator (SpeedVac, Thermo), 100 µL Mox reagent (2% methoxyamine in pyridine, Thermo) was added to the 1.5-mL tubes, and the metabolites were methoxylated at 30°C and 1,200 rpm for approximately 6 h using a Thermo shaker (BSR-MSC100, Biomedical Sciences). After methoxylation, 50 µL (1% v/v) of trimethylchlorosilane (TMS, Thermo) were added to the 1.5-mL tubes. For TMS derivatization, the mixture was incubated for 30 min at 1,200 rpm and 37°C, as stated above. Finally, 50 µL of the derivatized samples were dispensed in vials for wide-target metabolome analyses of GC-QqQ-MS (AOC-5000 Plus with GCMS-TQ8040, Shimadzu Corporation). Raw data collection was performed using the GCMSsolution software (Shimadzu Corporation). Calculation of the peak area values was conducted using MRMPROBS (Tsugawa et al., 2013; Tsugawa et al., 2014a; Tsugawa et al., 2014b). Peak areas were normalized using a quality control sample and the LOWESS/Spline normalization tool (Tsugawa et al., 2014a). Detailed GC-QqQ-MS parameters and MRM transitions for wide-target analysis were previously described (Tabeta et al., 2021; Tabeta et al., 2022).

### Statistical analyses

Statistical analyses were performed using Tukey’s HSD test (R ver. 4.0.2.; R Core Team, 2020). Multiple comparisons were performed using the multcomp package (Hothorn et al., 2008). PCA plots were calculated and constructed using Statistics in Microsoft Excel (URL http://prime.psc.riken.jp/compms/others/main.html#Statistics) (Tsugawa et al., 2015; Matsuo et al., 2017). RT-qPCR data were analyzed using the randomization method from the Relative Expression Software Tool REST2009 (Pfaffl et al., 2002) as in Hanchi et al. (2018).

## Results

### Effect of the *pecp2* and *vhp2;1 vhp2;2* mutations on cotyledon gross morphology and cellular phenotypes

**Figure 2:**
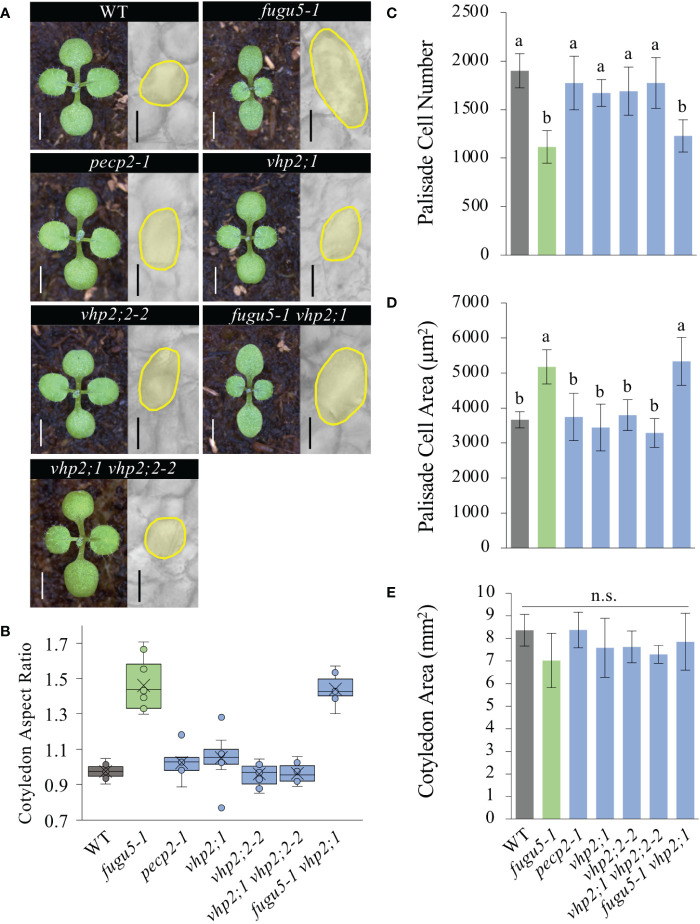
Quantification of cotyledon morphological and cellular phenotypes. **(A)** Gross morphology of the indicated genotypes (left), grown on rockwool for 10 DAS. Scale bar = 2 mm. Micrographs of palisade tissue cells from cleared cotyledons of WT, *fugu5-1*, *pecp2-1*, *vhp2;1*, *vhp2;2-2*, *vhp2;1 vhp2;2-2* and *fugu5-1 vhp2;1* lines (right), at 25 DAS. Scale bar = 50 µm. **(B)** Box plots of cotyledon aspect ratio at 25 DAS (*n* = 8). The lower and upper portions of the boxes are the first and third quartiles, respectively, and the dividing line is the median. Dots represent each value. **(C–E)** Numbers of subepidermal palisade tissue cells **(C)**, their average sizes **(D)**, and the average sizes of each cotyledon **(E)** at 25 DAS. Data are means ± SD (*n* = 160 cells from eight different cotyledons). Each character represents a significant difference at *P*< 0.05 (Tukey’s HSD test). n.s., not significant. DAS, days after seed sowing.

The loss of H^+^-PPase activity in *fugu5* resulted in excess PPi and diminished sucrose production from seed storage lipids (Ferjani et al., 2011). Careful examination revealed that postgerminative reactivation of cell cycling, particularly in the mediolateral axis of cotyledons, was compromised in the *fugu5* mutants, which apparently led to their typical oblong shape (Ferjani et al., 2007; Ferjani et al., 2011; **Figure 2A**). To examine this more closely, cotyledon morphology was assessed by quantification of their aspect ratio at maturity, namely at 25 DAS. These findings confirmed our previous results, as the *fugu5-1* aspect ratio was ~1.5; in comparison, the WT aspect ratio was around 1.0 (**Figure 2B**). In addition, whereas the *pecp2-1*, *vhp2;1*, *vhp2;2-2*, and *vhp2;1 vhp2;2-2* aspect ratios were comparable to the WT, *fugu5-1 vhp2;1* cotyledons were reminiscent of *fugu5-1* (**Figure 2B**). Together, these results indicate that the *pecp2-1*, *vhp2;1*, *vhp2;2-2*, and *vhp2;1 vhp2;2-2* mutations do not affect cotyledon shape, and that cotyledons are oblong only in the *fugu5* background.

*fugu5* mutants were originally characterized as having cotyledons that contain fewer but larger cells, a phenotype that we have named compensation (Ferjani et al., 2007; Ferjani et al., 2008). Although cotyledons of the *pecp2-1*, *vhp2;1*, *vhp2;2-2*, and *vhp2;1 vhp2;2-2* mutations did not differ morphologically, they may have differences at the cellular level, despite their apparent similarities. Quantitative analysis of cotyledon area, palisade cell number and cell size in cotyledons of the WT and the above genotypes revealed that only *fugu5-1* and *fugu5-1 vhp2;1* exhibited compensation, whereas the other mutant lines were indistinguishable from the WT (**Figures 2C–E**). Note that the mutants and the transgenic lines did not show any remarkable phenotypes in later developmental stages.

### Effect of the *pecp2* and *vhp2;1 vhp2;2* mutations on pavement cell shape and stomata distribution

The cotyledon epidermis in Arabidopsis is composed of two types of cells: the pavement cells (PCs) and the stomata. In the *fugu5* mutant backgrounds, excess PPi reduced PC complexity and altered stomatal distribution (Gunji et al., 2020). It is worth noting that while externally supplied sucrose suppressed compensation in the palisade tissue, the above epidermal developmental defects did not recover (Gunji et al., 2020). Hence, the above key phenotypes can be used as indicators of excess PPi in the epidermis of a given Arabidopsis plant, irrespective of its genotype.

**Figure 3:**
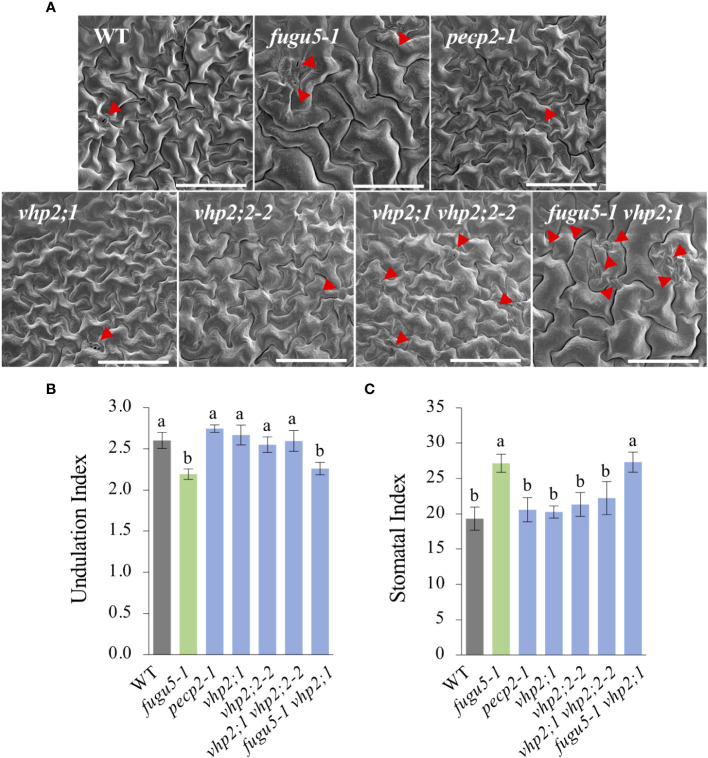
Quantification of pavement cell complexity and stomata distribution on the cotyledon adaxial side. **(A)** Representative SEM images of pavement cells (PCs) from the cotyledon adaxial side of WT, *fugu5-1*, *pecp2-1*, *vhp2;1*, *vhp2;2-2*, *vhp2;1 vhp2;2-2* and *fugu5-1 vhp2;1* lines at 25 DAS. Red arrowheads indicate stomata. Scale bar = 150 µm. **(B)** Undulation index (UI) of PCs. Data are means ± SD (*n* = 5 cotyledons). **(C)** Stomatal index (SI) determined as the number of stomata per 100 PCs. Data are means ± SD (*n* = 5 cotyledons). Each character represents a significant difference at *P<* 0.05 (Tukey’s HSD test). DAS, days after seed sowing.

Scanning electron microscope (SEM) images including the WT as a control indicated that whereas *fugu5-1* and *fugu5-1 vhp2;1* had simply-shaped PCs and abnormal stomatal distribution, PCs and stomatal distribution were apparently unaffected by the *pecp2-1*, *vhp2;1*, *vhp2;2-2*, or *vhp2;1 vhp2;2-2* mutations (**Figure 3A**). Subsequent quantification revealed that PC complexity and stomatal density, evaluated by the Undulation Index (UI) and the Stomatal Index (SI), respectively, were significantly different from the WT only in the *fugu5-1* background (**Figures 3B, C**).

### Impact of *PECP2* overexpression in the *fugu5* background on cotyledon morphological and cellular phenotypes

In our hands, the *pecp2* loss-of-function mutant did not display any of the excess PPi-related phenotypes previously recognized in the *fugu5* mutant, the master enzyme of PPi homeostasis (Ferjani et al., 2011; Ferjani et al., 2014; Segami et al., 2018). Therefore, to fully understand the impact of PECP2 regarding overall PPi levels *in planta*, we constructed transgenic lines constitutively overexpressing *PECP2* in the *fugu5-1* and *fugu5-3* mutant backgrounds. For convenience, lines constitutively overexpressing *PECP2* in the *fugu5-1* and *fugu5-3* backgrounds will be respectively referred to as *fugu5-1 35S::PECP2* A~C and *fugu5-3 35S::PECP2* A~C.

**Figure 4:**
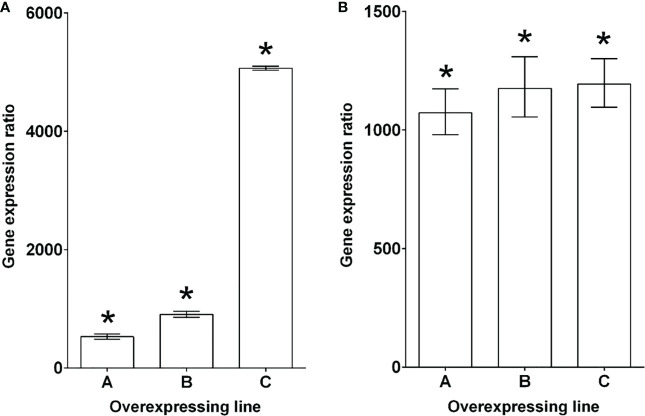
Characterization of *PECP2* overexpressing lines in *fugu5* mutant backgrounds. Lines that overexpress *PECP2* in the *fugu5-1* **(A)** and *fugu5-3* **(B)** backgrounds show very high transcript levels for these phosphatases, even in the high Pi condition. The results are expressed as gene induction level in overexpressing lines (#A to #C) in comparison to their respective *fugu5* controls grown under the same conditions. *fugu5* control values were arbitrarily set to one. The results are presented as the median ± 95% confidence interval. Asterisks indicate a statistically significant difference between overexpressing lines and *fugu5* (REST randomization test, *P*< 0.05). The reference genes used for the individual panels were *ROC3* and *GAPC1*.

**Figure 5:**
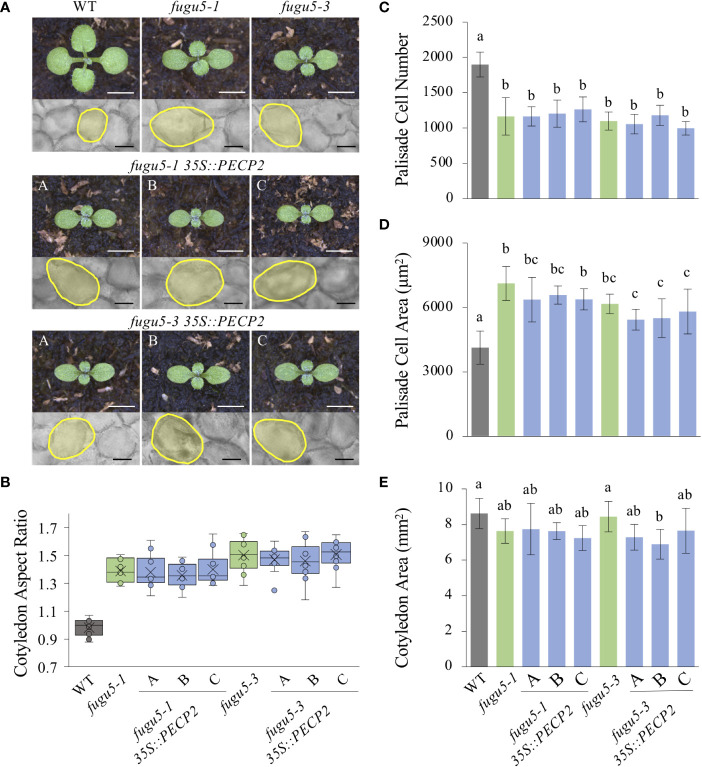
Quantification of cotyledon morphological and cellular phenotypes of *PECP2* overexpressing lines in the *fugu5* background. **(A)** Gross morphology of the indicated genotypes (above), grown on rockwool for 10 DAS. Scale bar = 2 mm. Micrographs of palisade tissue cells from cleared cotyledons of the WT, *fugu5-1*, *fugu5-3*, *fugu5-1 35S::PECP2*, and *fugu5-3 35S::PECP2* lines (below), at 25 DAS. Scale bar = 50 µm. **(B)** Box plots of cotyledon aspect ratio at 25 DAS (*n* = 8 cotyledons). The lower and upper portions of the boxes are the first and third quartiles, respectively, and the dividing line is the median. Dots represent each value. **(C–E)** Numbers of subepidermal palisade tissue cells **(C)**, their average sizes **(D)**, and the average sizes of each cotyledon **(E)** at 25 DAS. Data are means ± SD (*n* = 160 cells from eight different cotyledons). Each character represents a significant difference at *P*< 0.05 (Tukey’s HSD test). DAS, days after seed sowing.

qRT-PCR quantification of *PECP2* mRNA level in *fugu5-1 35S::PECP2* lines A, B, and C indicated fold changes of 500, 900 and 5,000 in transcript level, respectively, in comparison to their *fugu5-1* controls (**Figure 4A**). *PECP2* mRNA was even more overexpressed in the *fugu5-3 35S::PECP2* lines in comparison to their *fugu5-3* controls (with fold changes of 1,100 for *line* A and ~1,200 for lines B and C (**Figure 4B**). If PECP2 shows a significant pyrophosphatase activity *in planta*, its constitutive overexpression in the above transgenic lines should restore excess PPi content to either normal or lower levels, and restore the developmental defects stated above. To formally check this, we assessed the cotyledon aspect ratio and palisade tissue cellular phenotypes in the WT, *fugu5-1*, and *fugu5-3* lines, as well as the above transgenic lines (**Figure 5**). Overall, the constitutive overexpression of *PECP2* in both *fugu5-1* and *fugu5-3* had no remarkable effect on morphological or cellular phenotypes. More specifically, neither cotyledon shape (**Figures 5A, B**) nor compensation (**Figures 5C–E**) phenotypes were restored in spite of the extremely high expression level of *PECP2*. Taken together, these results imply that PECP2 is unlikely to act as an efficient PPi-scavenging enzyme *in planta*, and that the findings of May et al. (2011) might be reflecting particular *in vitro* conditions.

**Figure 6:**
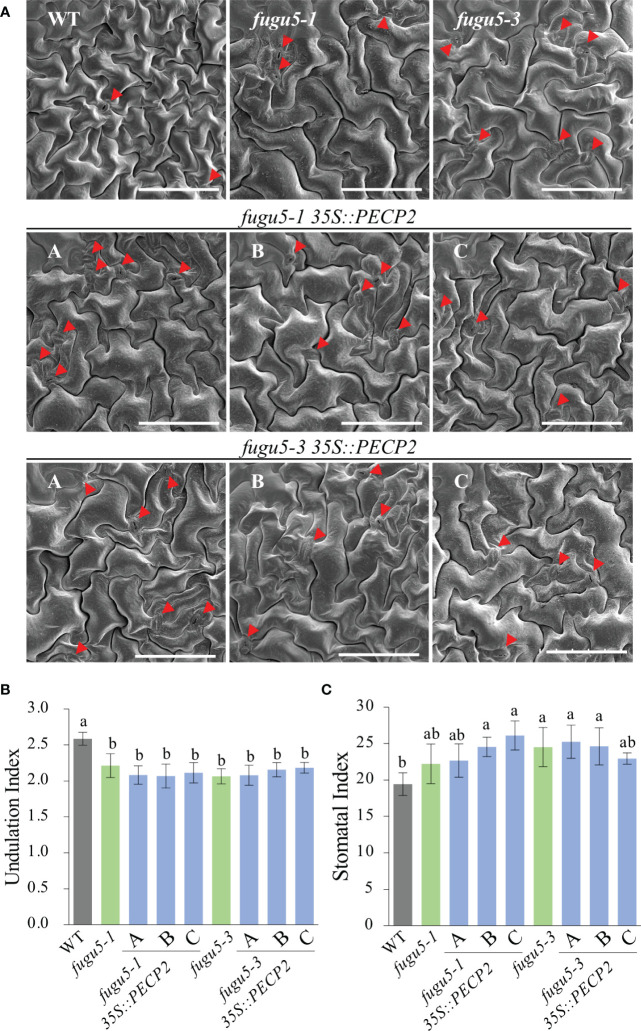
Quantification of pavement cell complexity and stomata distribution on the cotyledon adaxial side in *PECP2* overexpressing lines in the *fugu5* background. **(A)** Representative SEM images of pavement cells (PCs) from the cotyledon adaxial side of the WT, *fugu5-1*, *fugu5-3*, *fugu5-1 35S::PECP2*, and *fugu5-3 35S::PECP2* lines at 25 DAS. Red arrowheads indicate stomata. Scale bar = 150 µm. **(B)** Undulation index (UI) of PCs. Data are means ± SD (*n* = 5 cotyledons). **(C)** Stomatal index (SI) determined as the number of stomata per 100 PCs. Data are means ± SD (*n* = 5 cotyledons). Each character represents a significant difference at *P<* 0.05 (Tukey’s HSD test). DAS, days after seed sowing.

Next, the transgenic lines were used to evaluate the consequences of the constitutive overexpression of *PECP2* on PC shape, stomatal density and distribution. We found that while the WT PCs were complex (UI ≈ 2.5), UI values in *fugu5-1*, *fugu5-3*, and all transgenic lines were significantly reduced (UI ≈ 2.0) as compared to the WT (**Figures 6A, B**). In addition, SI values clearly indicated that stomata number was consistently higher in the *fugu5* background, including the transgenic lines, and SEM images indicated that stomata density was also affected, mimicking *fugu5* single mutant phenotypes (**Figure 6C**; Gunji et al., 2020). Together, the above results demonstrate that constitutive overexpression of *PECP2* failed to rescue the epidermal developmental defects in the *fugu5* background. If we assume that the anatomical phenotypes are PPi-specific (Gunji et al., 2020; Gunji et al., 2022), the above data also suggest that constitutive overexpression of *PECP2* failed to restore PPi levels.

### Impact of *PECP2* overexpression in the *fugu5* background on the skotomorphogenic developmental program

**Figure 7:**
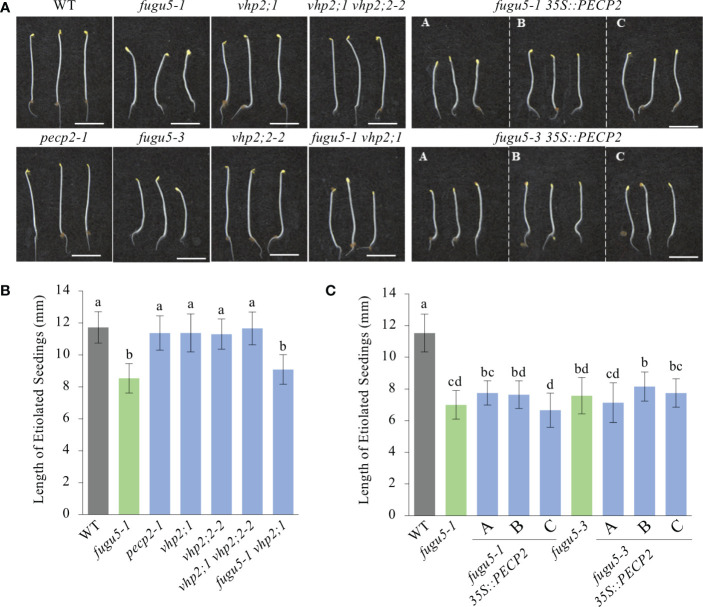
Quantification of hypocotyl length of etiolated seedlings. **(A)** Photographs of etiolated seedlings of the indicated genotypes grown on MS-only medium (without sucrose) in the dark for 4 DAI. Scale bar = 5 mm. **(B)** Hypocotyl length of etiolated seedlings of the WT, *fugu5-1*, *pecp2-1*, *vhp2;1*, *vhp2;2-2*, *vhp2;1 vhp2;2-2* and *fugu5-1 vhp2;1* lines grown on MS-only medium in the dark for 4 DAI. Data are means ± SD (*n* = 20 seedlings). **(C)** Hypocotyl length of etiolated seedlings of the WT, *fugu5-1*, *fugu5-3*, *fugu5-1 35S::PECP2* and *fugu5-3 35S::PECP2* lines grown on MS-only medium in the dark for 4 DAI. Data are means ± SD (*n* = 20 etiolated seedlings). Each character represents a significant difference at *P<* 0.05 (Tukey’s HSD test). DAI, days after induction of seed germination.

Arabidopsis seedlings grown in the dark follow a skotomorphogenic developmental program, whereby seed reserve resources are allocated toward hypocotyl elongation at the expense of other organs, namely cotyledons and roots (Josse and Halliday, 2008). Upon seed imbibition, dark-grown seedling establishment depends on oilseed mobilization as the sole source of energy, until photosynthesis begins. It is worth mentioning that the lack of sucrose in *fugu5-1* etiolated seedlings, due to the inhibitory effect of PPi on gluconeogenesis, reduced hypocotyl length by ~30% as compared to the WT (Ferjani et al., 2011; Katano et al., 2016). Therefore, to evaluate the role of PECP2, VHP2;1 and VHP2;2 in this process, hypocotyl length was determined by using all genotypes indicated in **Figure 7**.

Our measurements clearly indicated no impact on the hypocotyl length of either single mutants (i.e. *pecp2-1*, *vhp2;1*, and *vhp2;2-2*) or double mutants (*vhp2;1 vhp2;2-2*; **Figures 7A, B**). Moreover, while *fugu5-1* and *fugu5-3* hypocotyls were significantly shorter (**Figures 7A, B**) in comparison to the WT, constitutive overexpression of *PECP2* did not rescue hypocotyl elongation defects (**Figures 7A–C**). Altogether, these findings are in line with our aforementioned conclusions that the PECP2, VHP2;1 and VHP2;2 enzymes do not strongly influence overall PPi levels in plant cells *in vivo*.

### Gluconeogenesis from seed storage lipids is not affected in the *pecp2-1*, *vhp2;1*, or *vhp2;2-2* mutant backgrounds

In Arabidopsis, defects in V-PPase activity lead to a net increase in PPi content (up to ~2.5-fold), partial inhibition of the gluconeogenic enzyme UGPase (Ferjani et al., 2018), and a 50% reduction in sucrose concentration (Ferjani et al., 2011). These reports indicate that PPi homeostasis is important during postgerminative development, and that UGPase is rate-limiting (Park et al., 2010). Although PECP2 has been reported as being one of the rare PPase enzymes belonging to the HAD superfamily (Lee et al., 2009; May et al., 2011), the quantification of morphological traits has failed to support its role as an enzyme able to efficiently hydrolyze PPi once in the cell context.

**Figure 8:**
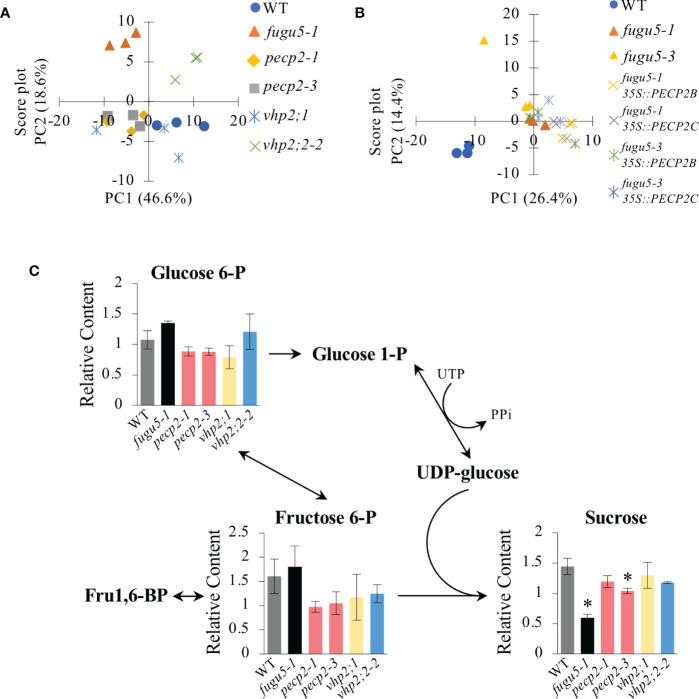
Comparative metabolomic analysis of etiolated seedlings. Metabolomic analysis of etiolated seedlings was performed by GC-MS/MS (*n* = 3 biological replicates; 100 etiolated seedlings per sample). Etiolated seedlings were grown on MS-only medium in the dark for 3 DAI. **(A)** Principal Component Analysis (PCA) score plot of the WT, *fugu5-1*, *pecp2-1*, *vhp2;1*, *vhp2;2-2*, *vhp2;1 vhp2;2-2* and *fugu5-1 vhp2;1* lines. **(B)** PCA score plot of the WT, *fugu5-1*, *fugu5-3*, *fugu5-1 35S::PECP2* and *fugu5-3 35S::PECP2* transgenic lines. Color plots refer to the genotype in the legend. **(C)** Pathway analysis focusing on gluconeogenesis-related metabolites. Metabolite contents relative to the quality control sample are plotted on the corresponding pathway. Data are means ± SD (*n* = 3 biological replicates; 100 etiolated seedlings per sample). Asterisks indicate mutants with significant differences as compared to the WT (Dunnett’s test at *P*< 0.05). DAI, days after induction of seed germination.

In order to assess the role of PECP2 and VHPs in PPi homeostasis, we performed a comparative metabolomic analysis at 3 DAI by using GC-MS-MS to detect any subtle metabolic perturbation in these mutant lines. First, metabolomic data were subjected to Principal Component Analysis (PCA), which provides a more comprehensive view of patterns in metabolic profiles among genotypes (Ferjani et al., 2018; Gunji et al., 2022). The PCA (**Figure 8A**) first component (PC1) explained 46.6% of the metabolic variation, and the second component (PC2) explained 18.6%. Collectively, PC1 and PC2 depicted 65.2% of the total variance among genotypes. PC1 showed scattered plots and identified several differences in metabolic profiles. Whereas the WT, *pecp2-1*, *pecp2-3*, *vhp2;1* and *vhp2;2-2* lines belonged to the same group, *fugu5-1* showed a remarkable difference along PC2 (**Figure 8A**).

In contrast, the overexpression of *PECP2* in the *fugu5-1* or *fugu5-3* background had a negligible effect on their metabolic profiles in comparison to the parental lines, which showed a clear separation between the WT and *fugu5 35S::PECP2* lines along PC1 (**Figure 8B**). Finally, pathway analysis focusing on gluconeogenesis revealed that the contents of major metabolites of the central metabolism, previously recognized to be up- or down-regulated in *fugu5* due to excess PPi, were unaffected (**Figure 8C**). Taken together, our comparative metabolomic analysis, performed using highly sensitive GC-MS-MS, confirms that PECP2 and VHPs show no signs of cytosolic PPi homeostasis defects *in planta*.

## Discussion

In this study, our careful in-depth phenotypic characterization led us to the conclusion that PECP2, VHP2;1 and VHP2;2 have a negligible impact on the overall cytosolic PPi levels *in vivo*. In the process, we established a set of different parameters that can be used as references for the identification of PPi homeostasis defects at different developmental stages of the plants. By quantifying the cotyledon aspect ratio, palisade tissue cellular phenotypes, adaxial side pavement cell complexity, stomatal distribution, and etiolated seedling length, especially when used in combination with *fugu5* reference mutants, we were able to reveal subtle alterations of cytosolic PPi levels using anatomical alterations as a proxy for PPi quantification. This strategy is reminiscent of the successful screens for mutants impaired in Pi homeostasis that were based on observed defects in root hair elongation or density, primary root growth, or anthocyanin development (Williamson et al., 2001; Reymond et al., 2006). Considering the difficulties of direct PPi quantification, which is considerably more complex than Pi quantification, the possibility to use PPi-related anatomical defects at different developmental stages could be instrumental in identifying new genes involved in the regulation of this important metabolite.

In the case of the VHP2;1 and VHP2;2 proteins, their lack of a general impact on PPi homeostasis might be due to their overall low expression levels, since protein quantification of these type-II H^+^-PPases revealed that they amount to only ~0.2% of type-I H^+^-PPase levels (Segami et al., 2010). Low expression levels might actually be linked with their presence within the Golgi membrane, as opposed to the tonoplastic type-I H^+^-PPases: the cells maintain a sharp pH gradient of about Δ2 pH units between the cytoplasm and the acidic vacuole, while a Δ0.5-1 pH unit is sufficient to maintain the slightly more acidic cis-Golgi and TGN compartments when compared to the cytosol (Shen et al., 2013; Tsai and Schmidt, 2021). For this reason, the lack of *fugu5*-type phenotypes in *vhp2* mutants might not be a reflection of a lesser role for type-II versus type-I H^+^-PPases, but simply the consequence of a more limited proton pump activity required to maintain Golgi acidification. Generating overexpressing lines in a *fugu5* background, as we did here with *PECP2* overexpressers, might be used to validate this hypothesis. Type-II H^+^-PPases remain a very intriguing area of study, due to their specific Golgi targeting (as opposed to the vacuolar location of FUGU5/type-I H^+^-PPases; Drozdowicz and Rea, 2001; Mitsuda et al., 2001; Segami et al., 2010). Nevertheless, the respective importance of their combined functions (PPi hydrolysis coupled to proton pumping) still awaits demonstration of their impact on biological functions. As deletion of these transporters has no overall impact on PPi homeostasis, it will be important to characterize the impact of *vhp2* mutations on Golgi pH maintenance, likely in combination with mutations of other known membrane-bound pH regulators present in the endomembrane network (McKay et al., 2022). Overall, a careful analysis of these mutants, particularly under stress conditions where PPi could be limiting, and where pH maintenance within the Golgi might be affected, is necessary to understand under which conditions the dual activity of these proteins (pyrophosphatase/proton pump) could become limiting.

For PECP2, we provided a more complete demonstration that this enzyme, although initially shown to be a pyrophosphatase based on *in vitro* data (May et al., 2011), actually does not significantly impact PPi homeostasis *in planta*. This was already suggested following the characterization of hypocotyl elongation in *pecp1/2/3* triple mutants and *fugu5 pecp1/2* triple mutants (Hanchi et al., 2018), but we performed a more in-depth characterization of these mutants, along with the demonstration that *fugu5* PPi homeostasis defects cannot be complemented by *pecp2* overexpression. The initial *in vitro* characterization of PECP2 (May et al., 2011), completed later on by characterizing PECP1 and PECP3 (May et al., 2011; Hasnain et al., 2016; Tannert et al., 2021), remains fundamental for the study of the PECP family, as it clearly demonstrates that each of these enzymes can dephosphorylate a range of different substrates with different affinities. For instance, each PECP protein demonstrates some ability to cleave PPi, even if only marginally. In fact, the pyrophosphatase activity measured *in vitro* for PECP2 was quite low (<1 nmol Pi min^-1^ mg protein^-1^; May et al., 2011) in comparison to the one associated with PECP3 (177 nmol Pi min^-1^ mg protein^-1^; Tannert et al., 2021), although another study only revealed a very marginal pyrophosphatase activity for PECP3 (Hasnain et al., 2016). Interestingly, their *in vitro* specific activities also suggested that each of these three enzymes metabolizes PEtn more efficiently than PCho *in vitro* (PECP1: 3,665 (PEtn) versus 475 (PCho) nmol Pi min^-1^ mg protein^-1^ (May et al., 2012); PECP2: 0.02 (PEtn) versus<0.001 (PCho) nmol Pi min^-1^ mg protein^-1^ (May et al., 2011); PECP3: 1,379 (PEtn) versus 315 (Pcho) nmol Pi min^-1^ mg protein^-1^ (Tannert et al., 2021). Furthermore, PECP3 was initially described as being a thiamine monophosphatase (ThMP) *in vitro* (Hasnain et al., 2016), but direct comparison with PEtn and PCho revealed that its specific activity towards ThMP is actually intermediate (895 nmol Pi min^-1^ mg protein^-1^; Tannert et al., 2021).

Substrate affinities and expected substrate concentrations must also be taken into account before predicting *in vivo* functions, even though the comparison of enzyme-specific activities already provides an interesting quantification of the enzymatic dephosphorylation capabilities. Nevertheless, characterization of mutants and overexpressing lines constitute a necessary step for the effective determination of the *in vivo* substrates of HAD-type proteins such as Arabidopsis PECP1/2 and 3. Although a whole range of molecules have been described as potential substrates for these enzymes, including primarily PEtn and PCho for PECP1, PPi for PECP2, and ThMP for PECP3 (May et al., 2011; Hasnain et al., 2016), only PEtn and PCho homeostasis were *de facto* altered in mutant and overexpressing lines (Hasnain et al., 2016; Mimura et al., 2016; Hanchi et al., 2018; Tannert et al., 2018; Angkawijaya et al., 2019; Tannert et al., 2021). Even though there is some discrepancy regarding whether PEtn or PCho could be considered as a favored substrate of these enzymes, all *in planta* data (including this study) point to a role for this enzyme family in PEtn and PCho dephosphorylation. PECP2 is therefore unlikely to play a major role in cytosolic PPi homeostasis *in vivo.*

The impact of this protein could possibly be limited to its subcellular compartment, and indeed it has been suggested that PECP1 and 2 associate with ER structures through GFP-tagging (Angkawijaya et al., 2019). It might be relevant to refine and confirm this localization through complementary methods to eliminate the risk of artefacts associated with GFP-tagging strategies, or to identify the enzyme action site. Even so, considering that changes in the expression level of *PECP2* had a very a strong impact on PEtn and PCho content at the whole-cell level, it is unlikely that the observed lack of a PPi defect might be caused by PECP2 association with the ER.

Discrepancies between the *in vitro* and *in vivo* data should certainly not discourage further *in vitro* characterization of HAD-type proteins. However, these gaps are a reminder that the range of substrates assayed *in vitro* must be as large as possible and that ultimately, the cell environment can strongly affect the enzyme activities. *In vitro* assays on purified PECP2 proteins (May et al., 2011) were performed using substrate concentrations that could occur *in vivo*, as candidate substrates were added at a concentration of 500 µM. The PPi concentration is estimated to be within that range *in vivo*, as it is maintained around 0.2-0.3 mM in the cytosol (Weiner et al., 1987). Using PCho at 500 µM is also reasonable *in vitro*, since PCho and PPi contents in plants are within the same range (around 50 nmol/g FW; Heinonen, 2001; Kraner et al., 2017).

However, it is rarely possible to test a mix of candidate substrates to evaluate their possible competition, since the enzyme activity assays are often based on the measurement of phosphate release (typically quantified by ammonium molybdate or malachite-based assays). As the output (i.e. phosphate release) is common to all cleaved substrates, this method is not applicable to the study of substrate competition. In addition, the activity of all PPases (membrane-bound as well as soluble and HAD-like proteins) is strongly dependent on the nature and quantity of metal ions available to the purified protein (May et al., 2011; Kuznetsova et al., 2015; Grzechowiak et al., 2019; Tsai et al., 2019). As such, assay conditions might not correspond to *in vivo* metal content.

Altogether, *in vitro* properties will only reflect the behavior of enzymes within the limits of the artificial conditions selected for the assays. This might only be remotely related to *in vivo* conditions, once the mix of ion metals and substrates competing for the candidate protein in the cytoplasm of plant cells is taken into account. These knowledge gaps also demonstrate the limits of our understanding of the key determinants of HAD-type protein-substrate recognition specificities, promoting active research in this field (Tian et al., 2022).

## Data availability statement

The original contributions presented in the study are included in the article/**Supplementary Material**. Further inquiries can be directed to the corresponding authors.

## Author contributions

AF conceived the project and designed, supervised, and funded the study. HTo performed the experiments, conducted the phenotyping analysis, and analyzed the data. HTa performed wide-target metabolomics, data collection and analyzed data. HTo and SG performed SEM observations and the quantification of all epidermis phenotypes. HJ generated the overexpressing lines and analyzed the predicted protein structures. PD performed the qPCR experiments. MH supervised and funded the study. HJ and AF wrote the paper with input from the coauthors. All authors contributed to the article and approved the submitted version.

## Supplementary material

Supplemental Movie 1 Animation showing the overlay of selected type II H^+^-PPases. The overlay compares the predicted structures for FUGU5 (UniProt P31414; in bright green),VHP2;1 (UniProt Q56ZN6; in dark green) and VHP2;2 (UniProt Q9FWR2; in blue) to the crystal structure of *Vr*H^+^-PPase (PDB reference 4A01, Chain A; in light gray). A general overview of the 4 structures shows the strong conservation of these 16-TM proteins, with the exception of an additional N-terminal domain in VHP2;1 and 2;2, briefly shown in black. Subsequently, a rotated view zooms to compare the positions of 3 AA involved in PPi binding in *Vr*H^+^-PPase, with the corresponding positions within the 3 predicted structures of FUGU5, VHP2;1 and VHP2;2. Finally, a side view highlights the positions of 2 AA involved in proton pumping in *Vr*H^+^-PPase, overlapping with the corresponding positions within the 3 predicted structures of FUGU5, VHP2;1 and VHP2;2. AA: amino acid.
